# Prevalence, Seroprevalence and Risk Factors of Avian Influenza in Wild Bird Populations in Korea: A Systematic Review and Meta-Analysis

**DOI:** 10.3390/v15020472

**Published:** 2023-02-08

**Authors:** Eurade Ntakiyisumba, Simin Lee, Byung-Yong Park, Hyun-Jin Tae, Gayeon Won

**Affiliations:** 1 College of Veterinary Medicine, Jeonbuk National University, Iksan Campus, Gobong-ro 79, Iksan 54596, Republic of Korea; 2 Department of Veterinary Medicine and Bio-Safety Research Institute, Jeonbuk National University, Iksan 54596, Republic of Korea

**Keywords:** avian influenza virus, wild birds, prevalence, seroprevalence, systematic review, meta-analysis, South Korea

## Abstract

Since the first recorded outbreak of the highly pathogenic avian influenza (HPAI) virus (H5N1) in South Korea in 2003, numerous sporadic outbreaks have occurred in South Korean duck and chicken farms, all of which have been attributed to avian influenza transmission from migratory wild birds. A thorough investigation of the prevalence and seroprevalence of avian influenza viruses (AIVs) in wild birds is critical for assessing the exposure risk and for directing strong and effective regulatory measures to counteract the spread of AIVs among wild birds, poultry, and humans. In this study, we performed a systematic review and meta-analysis, following the PRISMA guidelines, to generate a quantitative estimate of the prevalence and seroprevalence of AIVs in wild birds in South Korea. An extensive search of eligible studies was performed through electronic databases and 853 records were identified, of which, 49 fulfilled the inclusion criteria. The pooled prevalence and seroprevalence were estimated to be 1.57% (95% CI: 0.98, 2.51) and 15.91% (95% CI: 5.89, 36.38), respectively. The highest prevalence and seroprevalence rates were detected in the Anseriformes species, highlighting the critical role of this bird species in the dissemination of AIVs in South Korea. Furthermore, the results of the subgroup analysis also revealed that the AIV seroprevalence in wild birds varies depending on the detection rate, sample size, and sampling season. The findings of this study demonstrate the necessity of strengthening the surveillance for AIV in wild birds and implementing strong measures to curb the spread of AIV from wild birds to the poultry population.

## 1. Introduction

Avian influenza (AI), also known as the “bird flu,” a disease caused by influenza type A viruses, affects a wide variety of domestic and wild birds. Based on their pathogenicity in birds, influenza A viruses are classified as either highly pathogenic or low pathogenic avian influenza viruses, known as HPAI and LPAI viruses, respectively [1,2]. Wild birds, particularly migratory aquatic birds of the order Anseriformes (ducks, geese, and swans) and Charadriiformes (shorebirds and gulls) are natural reservoirs of LPAI viruses [3,4,5]. As LPAI viruses primarily replicate in duck intestinal tracts, their transmission among wild birds occurs primarily through the fecal-oral route [1]. LPAI viruses are excreted in feces and have been demonstrated to survive in water for an extended period of time [6]. Thus, waterborne transmission could play a significant role in the spread of LPAI viruses among migratory waterbirds.

Generally, AI viruses do not cause disease in wild birds, although subtypes of HPAI viruses can invade and replicate in different organs and may cause severe infections [4,7]. HPAI viruses evolve by mutation when the virus, carried in its mild form by a wild bird, is introduced into poultry [5,8]. These viruses are capable of infecting a wide range of animal species, such as swine, birds, companion animals, marine animals, and humans [7,9]. The transmission of avian influenza viruses (AIVs) from infected wild birds to domestic birds is perceived to occur through the sharing of water sources or the contamination of feed [10,11]. In humans, zoonotic subtypes of AIVs are transmitted mainly through direct contact with infected domestic poultry [11,12]. To date, eight AIVs have been reported to infect humans, of which, the H5N1 and H7N9 subtypes are associated with high morbidity and mortality in a large number of humans [11,13,14]. In South Korea, the HPAI subtype, H5N1, was first detected in duck meat imported from mainland China in 2000, which resulted in the loss of 4588 tons of meat [15]. From 2003 to 2004, an HPAI outbreak affected 392 chicken and duck farms in South Korea, causing a total discard of 5,285,000 birds, which was equivalent to $458 million [15,16].

In wild birds, the first cases of the H5N1 HPAI virus infection were primarily observed in Hong Kong in late 2002 [17,18]. Since then, multiple AI outbreaks associated with the H5N1 subtype have been reported in Asia, Africa, and Europe, all of which have been ascribed to wild migratory birds [19,20]. These documented cases imply that wild aquatic birds may play a major role in carrying AIVs over long distances via migration. Waterfowl are the most observed migratory birds, and winter birds are predominantly associated with the occurrence of AI in South Korea [2,16]. Although many countries have been able to halt the spread of H5N1 in animal and human populations by conducting regular surveillance and enforcing strict animal health regulations, the virus remains endemic to poultry populations, primarily in low-income countries with inadequate animal health and surveillance facilities. Owing to the rapid evolution of HPAI viruses, their devastating impact on the global poultry industry, and the threat they pose to public health, it is critical to understand the prevalence of AIVs in wild birds for risk assessment and preparedness against future outbreaks.

The prevalence and seroprevalence of AIVs in the wild bird populations of South Korea have been reported in various individual studies; however, no attempt has been made to consolidate these studies to derive a robust prevalence estimate of AIVs using a meta-analytical approach. The crucial benefit of meta-analysis is that it combines evidence to achieve a more robust point estimate with a higher statistical power as compared with that obtained from a single study from where the data originated [21,22]. Currently, systematic reviews and meta-analyses are perceived as the best available knowledge sources to make decisions regarding treatment choices [23], and meta-analyses are broadly used to calculate precise estimates of disease frequency, such as disease incidence rates and prevalence proportions [21,24]. In various studies, meta-analysis and regression analysis techniques have been used to generate overall prevalence estimates of infectious agents in animal populations and provide empirical evidence on associated risk factors [11,25,26]. In this study, we performed a systematic review and meta-analysis to estimate the overall prevalence and seroprevalence of AI in wild birds, using data from available studies conducted in South Korea. We hypothesized that the detection rate of AI in wild birds would depend on the sampling period, detection method, sample size, and sample type. Thus, subgroup analysis was adapted to investigate the sources of heterogeneity between the reported prevalence from individual studies using the above-mentioned variables.

## 2. Materials and Methods

### 2.1. Study Design and Systematic Review Protocol

A systematic review and meta-analysis were performed in accordance with the Preferred Reporting Items for Systematic Review and Meta-Analysis Protocols (PRISMA-P) guidelines [27] to determine the prevalence and seroprevalence of AI in wild birds in South Korea (Table S1). The review question was structured in accordance with the “population, exposure, comparator, and outcome” (PECO) format. In this systematic review, the “population of interest” refers to the wild birds, and “exposure” refers to the AIVs. As this study is a systematic review and meta-analysis of prevalence, the category of “comparator” was not relevant to this study. The “outcomes of interest” included the detected prevalence and seroprevalence of AIVs in wild birds in South Korea.

### 2.2. Literature Search Strategy

An extensive literature search was conducted with no language restriction using MEDLINE (via PubMed), Scopus, Web of Science, and South Korean databases, such as RISS and KISS, to identify studies published between 1980 and 2021. The last literature search was conducted on 23 December 2021. The following keywords: (wild bird* OR migratory bird* OR waterfowl OR Galliformes OR Charadriiformes OR Anseriformes) AND (avian influenza* OR AI OR bird flu OR avian flu OR influenza A virus OR AIV) AND (Korea OR South Korea) AND (prevalence OR inciden* OR proportion OR cases OR surveillance OR seroprevalence) were used to find eligible studies on the prevalence and seroprevalence of AIVs in wild birds in South Korea. An asterisk was used to extend a search term to related words with the same meaning (e.g., inciden* for incidence and incident).

### 2.3. Eligibility and Exclusion Criteria

In this systematic review and meta-analysis, the inclusion criteria were as follows: cross-sectional studies, primary studies conducted in South Korea, studies that assessed the prevalence and/or seroprevalence of AI in wild birds, studies that reported the sample size and the number of positive samples or the prevalence/seroprevalence rate, and studies with virus-isolation data. Studies were excluded if they were not conducted in South Korea, if samples were collected from animals other than wild birds, and if they did not report the total number of samples alongside the number of positive samples detected or the exact calculated prevalence rate. The titles and abstracts were screened for suitability using predetermined criteria. The full texts of potentially relevant articles were obtained and evaluated.

### 2.4. Data Extraction

Data on the prevalence and seroprevalence of AI in wild birds in South Korea were extracted by two independent reviewers, and any disagreements were resolved through discussion and consensus. From all eligible studies, information regarding the first author, year of publication, publication status (i.e., published or non-published), sample type (i.e., feces, cloacal swabs, carcass, or blood), detection method, sampling season, sampling location, bird species, detected AI subtype, sample size, and the number of positive samples was extracted. Data were extracted and organized into a pre-developed Microsoft Excel spreadsheet.

### 2.5. Risk of Bias Assessment

The eligible studies were assessed for internal and external validity by two independent reviewers using the Joanna Briggs Institute (JBI) critical appraisal tools for prevalence studies [28,29]. Each study was classified as having a low, high, or unclear risk of bias. The checklist contained nine questions, but only eight were evaluated because one question (regarding the response rate) was irrelevant to this study.

### 2.6. Data Synthesis

Data analysis was conducted using R version 4.1.2 (R Studio version 1.4) software [30,31]. The meta-analysis was performed and the forest plots were generated using the “meta” and “metafor” packages [32,33,34]. The total number of samples collected and the number of positive samples detected in each study were used to calculate overall prevalence estimates. To fulfill the assumption of a normal distribution, the logit transformation method was applied to the data [24,26,35] using the following formula:

$$\begin{array}{c} logit\;p=\ln\left(\frac{p}{1-p}\right)\\ \mathrm{with}\;\mathrm{variance}:\;var\left(logit\;p\right)=\frac{1}{np}+\frac{1}{n\left(1-p\right)} \end{array}$$

where “n” is the total sample size and “*p*” is the prevalence of the pathogen under study. A generalized linear mixed model, together with a logit transformation, demonstrates better performance; different studies recommend the use of this approach, which was adapted in this study to pool the data [35,36]. A random effects model was used to generate the pooled prevalence and seroprevalence of AIV in wild birds in South Korea. To combine the study estimates, the between-study variance (τ^2^) was estimated using the maximum likelihood method. The overall effect size of the logit model and its corresponding 95% confidence interval (CI) were calculated and back-transformed to prevalence rates for ease of interpretation. The between-study heterogeneity was assessed using the Q test and *I*^2^ statistic, which accounts for the amount of the observed variance that reflects the variance in true effects rather than sampling error [37]. The heterogeneity between studies was considered substantially high if the Q test yielded a statistically significant *p*-value (*p* < 0.05) and *I^2^* was greater than 50%.

To investigate the reason for heterogeneity, a subgroup analysis was undertaken using four pre-specified variables, including sampling season (i.e., fall/winter and spring/summer), sample size (i.e., more than 1000 or less than 1000), sample type (i.e., feces, cloacal swabs, carcass, and blood), detection method (i.e., ELISA, reverse transcription-polymerase chain reaction (RT-PCR), rRT-PCR, hemagglutination (HA) test, virus isolation, hemagglutinin inhibition (HI) test, and agar gel precipitation test (AGPT)) that could potentially affect the reported prevalence in the literature. Publication bias was assessed through visual inspection of the symmetry of the contour-enhanced funnel plots, and a quantitative estimate of publication bias was performed using Egger’s regression test [38,39]. After confirming publication bias, the Duval and Tweedie trim-and-fill method was used to estimate an unbiased effect by imputing missing studies in the funnel plot [40].

## 3. Results

### 3.1. Search Results

**Figure 1:**
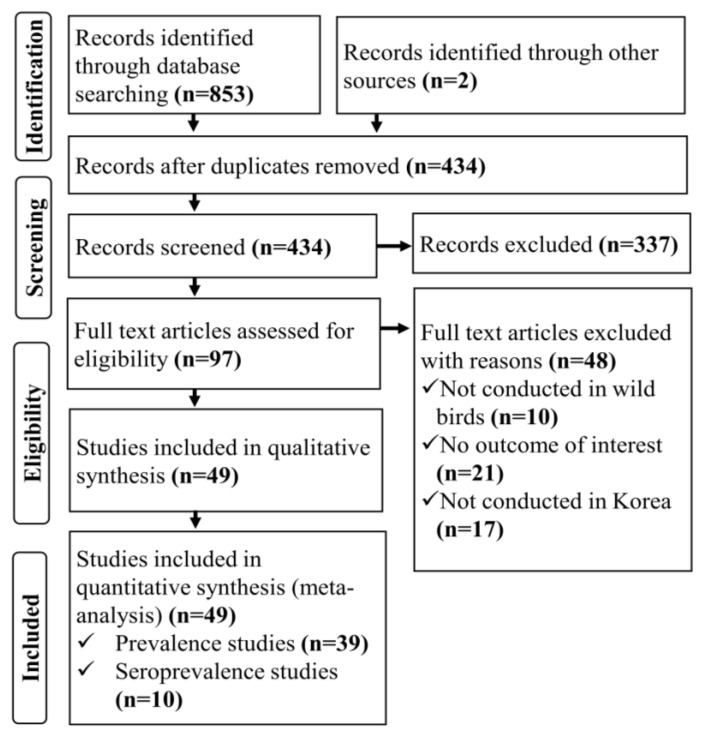
PRISMA flow diagram: Selection of studies on the prevalence and seroprevalence of avian influenza virus in wild bird populations in South Korea for use in a systematic review and meta-analysis.

Initially, 853 records were obtained by conducting an electronic database search. After duplicates were removed, 434 studies remained, and their titles and abstracts were reviewed for eligibility. After the title and abstract screening, 337 of the 434 records were removed. The remaining 97 studies were subjected to full-text screening, of which 48 were deemed irrelevant to this study, and the remaining 49 were finally included in the quantitative synthesis (meta-analysis). The study selection process is illustrated in Figure 1.

### 3.2. Study Characteristics

Among the 49 studies eligible for the meta-analysis, 39 assessed the prevalence and 10 assessed the seroprevalence of AIVs in wild birds in South Korea. Of the prevalence studies, 24 studies were published in peer-reviewed journals and 15 were non-published records (e.g., government reports, research institute reports, and student dissertations). Regarding the sampling season, 16 studies collected samples in fall and winter, whereas the other 23 studies did not report the sampling season. The sample types included feces (30 trials), carcasses (10 trials), cloacal swabs (10 trials), and combinations of samples (2 trials); two trials did not specify the type of samples used. Samples were collected from the Anseriformes (10 trials), Charadriiformes (5 trials), other species (9 trials), and non-reported bird species (36 trials). Regarding seroprevalence studies, three studies were published in South Korean or international academic journals, and the other seven were non-published records. Regarding the sampling season, three studies collected samples in the fall and winter, whereas the other seven studies did not report the sampling season. Blood samples were collected from the Anseriformes species (8 trials), Charadriiformes (5 trials), other species (8 trials), and non-reported species (3 trials). The characteristics of studies included in this systematic review and meta-analysis are summarized in Tables S2 and S3.

### 3.3. Risk of Bias Assessment

**Figure 2:**
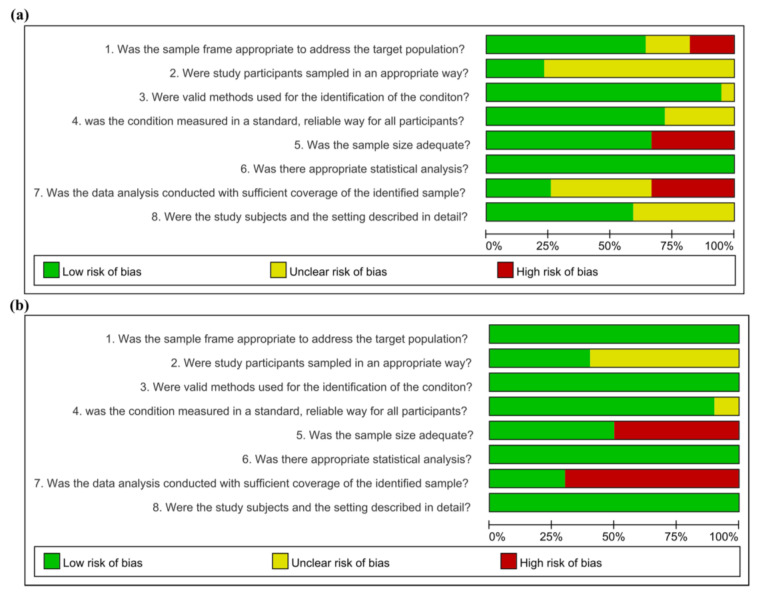
Risk-of-bias assessment of eligible studies on prevalence (**a**) and seroprevalence (**b**) of avian influenza viruses in wild birds of South Korea using the Joanna Briggs Institute (JBI) critical appraisal tools for prevalence studies.

The results of the quality assessment of relevant studies that reported the prevalence and seroprevalence of AIVs in wild birds are shown in Figure 2. Studies that reported both prevalence and seroprevalence had a risk of bias, assessed by sorting each result. Prevalence studies that used samples from the entire nation [8,17,41,42,43,44,45,46,47,48,49,50,51,52,53,54,55,56] or major migratory bird habitats [2,57,58,59,60,61,62,63] determined that the sampling frame was properly chosen, as samples were taken from within the pertinent regions to calculate the prevalence therein. The sampling frame made for the studies whose primary goal was to identify the characteristics of isolated AIVs [64,65,66,67] was judged to be at a high risk of bias because the isolation rate reported in the studies was constrained to a particular region. It was challenging to assume that the sample frame of the studies reporting the prevalence of carcasses referred for diagnosis was representative of the general wild bird population [68,69]. However, these prevalence studies used census, a suitable sampling method that examined all samples within a predetermined sampling frame. Seven primary studies that evaluated the prevalence of AIVs were judged to have a low bias in the sampling method, owing to proper capture methods from randomized wild birds [2,41,42,43,48,50,57]. All studies, except for two [59,66] that did not describe the isolation method in detail, recorded the condition of the samples by examining them with the proper techniques, such as RT-PCR [2,8,17,41,42,43,44,48,49,50,51,52,54,55,56,58,60,61,62,65,67,68,69,70,71,72,73,74], rRT-PCR [53,63,64,75,76], or HA tests [41,42,46,47,57,77]. All investigations, except for 12 studies [8,46,55,57,58,59,65,66,67,68,75,77], had read the experimental results with distinguishing criteria. Prevalence studies with small [56,57,58,59,64,65,66,67,68,69,71,73,74] and large sample sizes [2,8,17,41,42,43,44,45,46,47,48,49,50,51,52,53,54,55,60,61,62,63,70,72,75,76] were differentiated according to adequate sample size (1000 samples).

All studies unambiguously stated the number of examined samples and the number of positive or virus-detected samples. Studies that assessed prevalence in subgroups subdivided into sampling month [46,60,64,68,70,72], sampling year [2,8,48,49,51,54,55,72], province [17,41,42,43,51,58,59,60,68,75], and bird species [42,43,46,48,49,50,51,52,57,58,59,68,75] allowed for comparisons between the study sample and the population of interest. A low coverage bias was determined in prevalence studies [2,41,42,43,48,50,52,60,72,75] that used a similar sample number for each distinct subgroup. Otherwise, coverage bias was assessed as high [8,17,46,49,51,54,55,57,58,59,64,68,70].

Seroprevalence studies, in which samples were collected from the entire country [41,42,43,46,48,50,51] and major migratory bird habitats [2,49,78], were judged to set a suitable sample frame. Seroprevalence studies [2,41,42,43] that sampled random subjects using proper capture methods were judged to have low sampling bias. All experiments detected antibodies in serum using appropriate methods, including the HI assay [46,48,49] and ELISA [2,41,42,43,50,51,78]. All investigations, except for one [46], read the experimental results with distinguishing criteria. Seroprevalence studies with small [2,41,42,43,78] and large sample numbers [46,48,49,50,51] were differentiated according to adequate sample size (1000 samples). All the studies unambiguously stated the number of examined and positive samples. Studies that assessed prevalence into subgroups subdivided into sampling month [2], sampling year [2,48,51,78], province [43,49], and bird species [41,42,46,48,49,50,51,78] allowed for comparisons between the study sample and the population of interest. Low coverage bias was observed in prevalence studies [2,48,50] that used a similar sample size for each distinct subgroup. Otherwise, coverage bias was assessed to be high [41,42,43,46,49,51,78].

### 3.4. Meta-Analysis Results

#### 3.4.1. Prevalence Estimates

**Figure 3:**
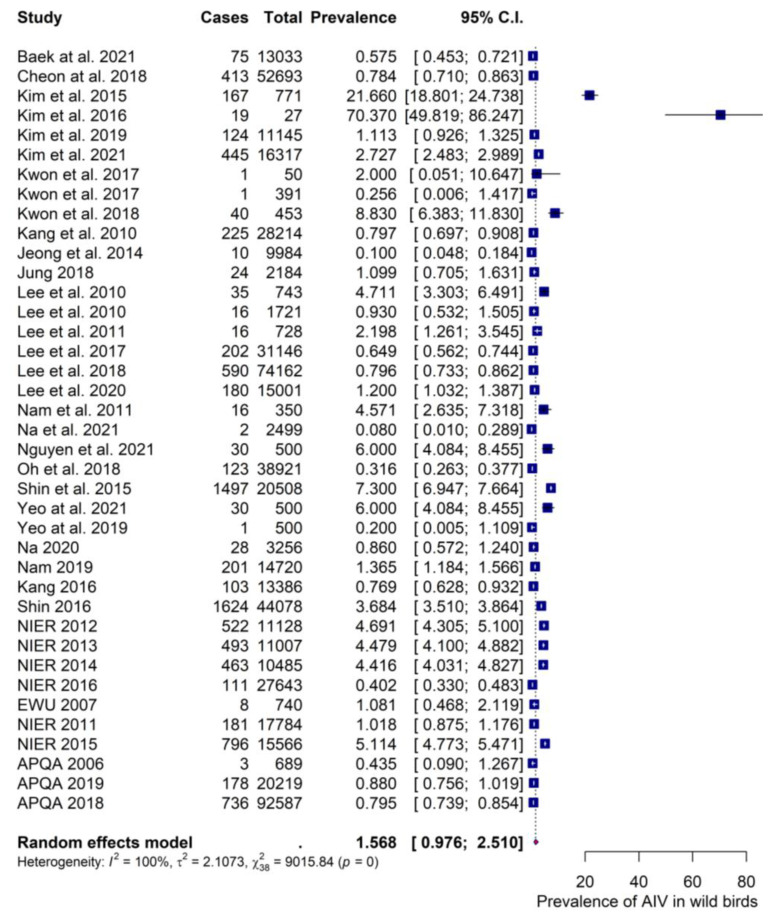
Forest plot of 39 studies assessing the prevalence of avian influenza virus in the wild bird populations of South Korea [2,8,17,41,42,43,44,45,46,47,48,49,50,51,52,53,54,55,56,57,58,59,60,61,62,63,64,65,66,67,68,69,70,71,72,73,74,75,76].

Thirty-nine studies investigated the prevalence of AIVs in wild birds in South Korea (Figure 3). Overall, the pooled prevalence was estimated to be 1.57% (95% CI: 0.98, 2.51) with high between-study heterogeneity (*I^2^* = 100%). Subgroup analyses were performed to investigate the source of heterogeneity using different variables that could potentially affect the prevalence rates among individual studies. Regarding bird species, the highest prevalence was detected in the Anseriformes species (4.34% (95% CI: 1.44, 12.30)), followed by a group of non-reported species (1.20% (95% CI: 0.74, 1.94)). The lowest prevalence was detected among Charadriiformes and other species (rather than Anseriformes and Charadriiformes), with a prevalence of 0.19% (95% CI: 0.03, 1.33) and 0.22% (95% CI: 0.04, 1.20), respectively. However, the heterogeneity was still high within the Anseriformes (*I^2^* = 98%) and non-reported (*I^2^* = 100) subgroups, whereas no heterogeneity was observed within the Charadriiformes and other species subgroups (*I*^2^ = 0 for each). Based on the sample type, the highest prevalence of 4.59% (95% CI: 0.76, 23.07) was detected in carcasses, followed by 1.58% (95% CI: 1.06, 2.36) and 1% (95% CI: 0.36, 2.75) in feces and cloacal swabs, respectively. The lowest prevalence, 0.63% (95% CI: 0.56, 0.71) and 0.97% (95% CI: 0.73, 1.28), was reported in mixed samples and non-reported sample types, respectively. The variables of sample size, sampling season, detection method, and publication status, had no significant influence on the prevalence rates (*p* > 0.05). All the variables assessed are presented in Table 1.

#### 3.4.2. Seroprevalence Estimates

**Figure 4:**
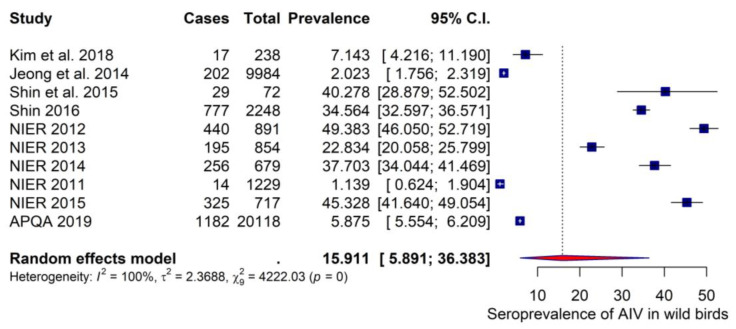
Forest plot of 10 studies assessing the seroprevalence of avian influenza virus in the wild bird populations of South Korea [2,41,42,43,46,48,49,50,51,78].

Ten studies assessed the seroprevalence of AIVs in wild birds in South Korea. The pooled seroprevalence estimate was 15.91% with a 95% CI of 5.89–36.38 (Figure 4). Between-study heterogeneity was significantly high (*I^2^* = 100%). To identify the reasons for heterogeneity, we conducted subgroup analysis using bird species, detection method, sample size, publication status, and sampling season as potential effect modifiers. All variables had a significant influence on seroprevalence rates, except for publication status (Table 2). Regarding bird species, the highest seroprevalence was detected in the Anseriformes species (30.45% (95% CI:18.97, 45.03)), followed by the Charadriiformes and non-reported species with seroprevalence estimates of 2.95% (95% CI: 0.24, 27.43)) and 2.85% (95% CI: 1.17, 6.76), respectively. The lowest seroprevalence was detected among other bird species (rather than Anseriformes and Charadriiformes), with an estimate of 2.83% (95% CI: 0.40, 17.26). Heterogeneity was still high within all subgroups (*I^2^* > 90%), except for the Charadriiformes species (*I^2^* = 49%). Based on the detection method, the highest seroprevalence, 31.47% (95% CI: 20.47, 45.02), was detected by ELISA, whereas the lowest seroprevalence of 2.46% (95% CI: 1.12, 5.31) was indicated by the HI test. The sample size also demonstrated a significant association with the seroprevalence rate, with the highest seroprevalence (30.93% (18.48, 46.93)) observed in studies with less than 1000 samples compared with 5.03% (1.25, 18.20) observed in those with more than 1000 samples (*p* < 0.01). Subgroup analyses also revealed that the highest seroprevalence was detected among studies that collected samples from fall to winter (36.48% (24.05, 51.01)) than in studies that did not report the sampling season (10.48% (3.46, 27.66)) (*p <* 0.02). Regarding the publication status, no significant difference in seroprevalence was observed between the published (9.07% (1.91, 33.78)) and non-published studies (19.85% (7.52, 43.01)) (*p <* 0.37). The results of the subgroup analysis are presented in Table 2.

### 3.5. Publication Bias

**Figure 5:**
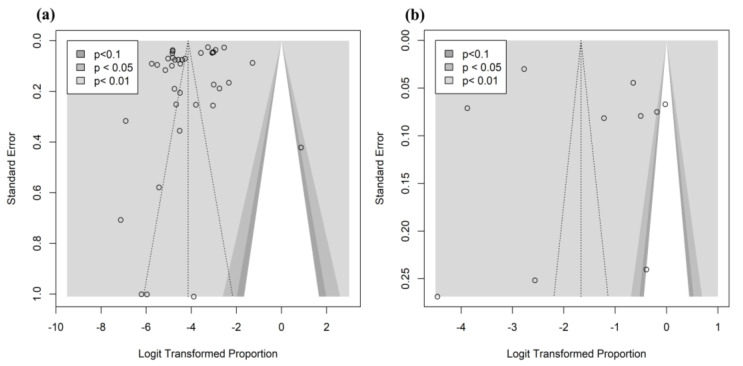
Contour-enhanced funnel plots for publication-bias assessment of the prevalence (**a**) and seroprevalence (**b**) of avian influenza virus in wild birds in South Korea.

Publication bias occurs when the likelihood of a study being published is influenced by its findings. In contrast to smaller studies with low effects, larger studies with relatively high effects are more likely to be published because they are statistically significant. This results in publication bias. To assess the presence of publication bias, contour-enhanced funnel plots were generated with the effect sizes on the *x*-axis and their standard errors on the *y*-axis (Figure 5). On visual inspection, the studies were symmetrically distributed on both sides of the mean effect and demonstrated significant results (*p* < 0.05). This symmetrical pattern suggests that a publication bias is unlikely. To avoid subjective inferences from funnel plot visualizations, Egger’s regression test was applied to quantify the presence of funnel plot asymmetry. Egger’s regression test yielded *p*-values of 0.094 and 0.506 for prevalence and seroprevalence outcomes, respectively, indicating no funnel plot asymmetry; hence, publication bias was not confirmed.

## 4. Discussion

AIVs in wild birds pose a pandemic threat to humans and the poultry industry worldwide. Previous studies have confirmed the relationship between the wild bird migratory route and AIV prevalence in South Korea by evaluating the geographical distributions of HPAI outbreaks and cases of mortality in wild birds [2,79]. Therefore, it is of critical importance to understand the current status of AI prevalence and seroprevalence in wild birds for use as an early warning system. In this study, we performed a systematic review and meta-analysis to consolidate the data from individual primary studies that evaluated the prevalence and seroprevalence of AIVs in wild birds in South Korea. The overall prevalence was estimated to be 1.568% (0.976; 2.510), indicating that approximately 2% of the wild bird population in South Korea are carriers of AIVs.

According to the census of winter migratory birds conducted in South Korea, approximately 1.63 million winter birds visited South Korea in 2020 [80]. Of these, 850,000 birds belonged to the order Anseriformes and accounted for 52% of the total. Based on the results of this meta-analysis, it can be estimated that approximately 32,600 migratory birds in South Korea carry AIVs. Chen et al. (2019) discovered that the prevalence and seroprevalence of AI were 2.5% and 26.5%, respectively, in wild birds in China [81]. The relatively low prevalence of AIVs in wild birds in South Korea is consistent with the knowledge that South Korea is not a breeding site, but rather a wintering area for adult wild birds, particularly waterfowl, such as ducks and geese [17]. On the other hand, the seroprevalence estimate was 15.911% (5.891; 36.383), suggesting that approximately 16% of the wild bird population in South Korea has been exposed to AIVs. As the antibody-positive cases included individuals that had recovered from AIV, the seroprevalence would tend to be relatively high compared with the prevalence. Another possible explanation for the high seroprevalence of AIVs in wild birds is that during the migration route, the migratory birds aggregate at nesting and feeding sites, which results in high rates of contact between birds, facilitating AIV transmission and a high prevalence of antibodies in the bird population [82,83]. It is, therefore, likely that wild birds arriving in South Korea will have had repeated exposure to AIVs, which leads to the persistence of anti-AIV antibodies over long periods in their bodies. The antibodies detected in wild birds could only be the result of seroconversion induced by a natural viral infection, as they are not immunized against AIV. Thus, they could play a critical role in spreading the virus to the surrounding environment, livestock, and humans.

Of the six variables used in the subgroup analysis, two (bird species and sample type) showed a significant influence on the prevalence rate of AIVs in wild birds. In contrast, four variables (bird species, detection method, sample size, and sampling season) showed a significant relationship with seroprevalence rates. Small studies (less than 1000 samples) demonstrated higher prevalence and seroprevalence rates than large studies (more than 1000 samples). This could be related to the fact that studies with small sample sizes are associated with higher effect sizes than bigger studies. Another possible reason is that the larger studies included in the analysis are mostly the non-published government reports that collected samples from different provinces of the country as part of a normal AIV surveillance routine, thus reducing the chance of getting positive samples compared with small studies that mainly collected samples from specific locations during or after an HPAI outbreak, thus increasing the probability of getting more positive samples. Regarding the species of wild birds, the highest AIV prevalence and seroprevalence rates were detected in the Anseriformes species compared to others. These results are in line with previous reports that waterfowl are the predominant migratory birds and are primarily associated with AI occurrence in South Korea [2,16]. Similar findings were also reported in China, where the highest AIV prevalence (6.8%) and seroprevalence (41.8%) were observed in the Anseriformes species compared with that in non-Anseriformes species [81]. Based on the sample type, the highest prevalence rate was revealed in carcasses compared to other sample types (*p* < 0.01). One possible reason for these results is that carcass samples were collected during or shortly after the 2014 HPAI outbreak (H5N8) in South Korean duck and chicken farms and wild birds found in the Donglim reservoir, Jeonbuk province [2,51,68,69]; most of these carcasses were confirmed to have died from the HPAI virus (H5N8) clade 2.3.4.6 [69,84].

Considering the detection method, the highest seroprevalence was detected by ELISA rather than the HI test (*p* < 0.01). This difference in performance could be due to the low sensitivity of the HI test for detecting AIV antibodies, particularly the H5N1 and H3N2 serotypes [85,86]. Furthermore, the highest seroprevalence rate detected during the fall-to-winter season is consistent with the National Institute of Environmental Research report “Surveillance and monitoring of wildlife diseases in Korea, 2012,” which states that the prevalence of AIVs increases from October to December (stage 1), when waterfowl migrate from the north, and in April (stage 4), when passing migratory birds are moving to the north [2]. Surprisingly, the results of the subgroup analysis confirmed no significant influence of the sampling season on the prevalence estimates. However, this should be interpreted with caution, as many studies included in the assessment of the prevalence did not clearly report the sampling season, and many studies fell into a subgroup of “not reported.” Consequently, this could have limited the power of the statistical tests to detect the significance while it was present. In addition to the above-mentioned moderators, prevalence rates in birds are likely to vary depending upon the surveillance period, the sampling region, and whether surveillance was performed in response to an outbreak or conducted as routine surveillance [11]. As most of the studies included in this meta-analysis did not provide clear information about these variables, we could not evaluate their contribution to the observed prevalence and seroprevalence rates. Although the prevalence and seroprevalence estimates between subgroups were significantly different, the within-subgroup heterogeneity was substantially high, indicating that none of the variables could entirely explain the reasons for between-study heterogeneity.

This study had a few limitations. First, there was substantial variation in the prevalence rates among individual studies. Although we used a couple of moderators to investigate the source of heterogeneity, only a few studies clearly reported on these variables, and a large number fell into the “not reported” subgroup. Furthermore, insufficient information was available to adequately categorize studies based on the reason for surveillance (in response to an outbreak or as routine surveillance), surveillance period, and sampling region, which are also relevant covariates that could possibly demonstrate significant relationships with the observed pooled prevalence estimates. Despite these limitations, the findings of this meta-analysis provide a more robust estimate of AIV prevalence and seroprevalence in wild birds in South Korea than that obtained from a single study.

## 5. Conclusions

In conclusion, this study provides solid evidence for the current prevalence of AIVs among the South Korean wild bird population. These findings demonstrated that a large number of wild birds in South Korea, particularly those of the order Anseriformes, are carriers of AIVs, and others have already been exposed to AI because of the high detection rate of anti-AIV antibodies. This poses a threat to the poultry industry and, potentially, to humans in South Korea, due to the critical role of wild birds in the spread of AIV. Furthermore, migratory wild birds have different flyways, which affect the distribution of AIVs in different countries. A multi-country surveillance system would provide detailed information on the prevalence and distribution of AIVs in this region. The evidence from this study highlights the need to strengthen existing preventive measures and increase surveillance activities to impede the risk of AIV transmission from wild birds to domestic poultry and human beings.

## Figures and Tables

**Table 1:** Results of subgroup analysis (prevalence rates) based on six potential effect modifiers.

Variables	Prevalence Estimates (95%)	*I*^2^ (%)	τ^2^	*P_subgroup_*
1. Bird species				<0.01 ^a^
Anseriformes	4.34 [1.44; 12.30]	98	3.2233	
Charadriiformes	0.19 [0.03; 1.33]	0	0	
Other species	0.22 [0.04; 1.20]	0	1.9568	
Not reported	1.20 [0.74; 1.94]	100	2.1236	
Overall prevalence	1.14 [0.72; 1.82]	99	2.8912	
2. Sample type				<0.01 ^a^
Feces	1.58 [1.06; 2.36]	99	1.2236	
Carcass	4.59 [0.76; 23.07]	95	7.1496	
Cloacal swabs	1.00 [0.36; 2.75]	100	2.6837	
Mixed samples	0.63 [0.56; 0.71]	0	0	
Not reported	0.97 [0.73; 1.28]	96	0.039	
Overall prevalence	1.7 [1.10; 2.64]	100	2.6417	
3. Detection method				=0.25 ^b^
RT-PCR	1.98 [1.11; 3.53]	100	2.3799	
HA-test	1.06 [0.59; 1.90]	95	0.5665	
rRT-PCR	0.75 [0.32;1.75]	85	0.8644	
Virus isolation	1.25 [0.14; 10.25]	88	2.0493	
Overall prevalence	1.54 [0.99; 2.38]	100	2.0266	
4. Sample size				=0.05 ^b^
Less than 1000	3.28 [1.20; 8.68]	97	3.3332	
More than 1000	1.10 [0.74; 1.65]	100	1.1152	
Overall prevalence	1.57 [0.99; 2.47]	100	2.1073	
5. Sampling season				=0.48 ^b^
Fall to winter	1.94 [0.80; 4.61]	98	3.1168	
Not reported	1.35 [0.82; 2.20]	100	1.4628	
Overall prevalence	1.57 [0.99; 2.47]	100	2.1073	
6. Publication status				=0.82 ^b^
Published	1.63 [0.80; 3.27]	100	3.07	
Non-published	1.47 [0.95; 2.28]	100	0.7476	
Overall prevalence	1.57 [0.99; 2.47]	100	2.1073	

^a^ The difference in prevalence estimates between subgroups was statistically significant. ^b^ There was no significant difference in prevalence estimates between subgroups.

**Table 2:** Results of subgroup analysis (seroprevalence rates) based on five potential effect modifiers.

Variables	Seroprevalence Estimates (95%)	*I*^2^ (%)	τ^2^	*P_subgroup_*
1. Bird species				<0.01 ^a^
Anseriformes	30.45 [18.97; 45.03]	100	0.7793	
Charadriiformes	2.95 [0.24; 27.43]	49	5.2003	
Other species	2.83 [0.40; 17.26]	94	7.0157	
Not reported	2.85 [1.17; 6.76]	99	0.6122	
Overall prevalence	7.71 [3.33; 16.86]	99	4.3612	
2. Detection method				<0.01 ^a^
ELISA	31.47 [20.47; 45.02]	97	0.5904	
HI test	2.46 [1.12; 5.31]	99	0.475	
Overall prevalence	15.90 [6.76; 33.01]	100	2.3668	
3. Sample size				<0.01 ^a^
Less than 1000	30.93 [18.48; 46.93]	98	0.7001	
More than 1000	5.03 [1.25; 18.20]	100	2.1246	
Overall prevalence	15.90 [6.76; 33.01]	100	2.3668	
4. Sampling season				=0.02 ^a^
Fall to winter	36.48 [24.05; 51.01]	98	0.2553	
Not reported	10.48 [3.46; 27.66]	100	2.53	
Overall prevalence	15.90 [6.76; 33.01]	100	2.3668	
5. Publication status				=0.37 ^b^
Published	9.07 [1.91; 33.78]	99	2.0361	
Non-published	19.85 [7.52; 43.01]	100	2.2482	
Overall prevalence	15.9 [ 6.76; 33.01]	100	2.2482	

^a^ The difference in seroprevalence estimates between subgroups was statistically significant. ^b^ There was no significant difference in seroprevalence estimates between subgroups.

## Data Availability

All data used or analyzed during this study are included in this article and its Supplementary Information Files.

## Supplementary Materials

The following supporting information can be downloaded at: https://www.mdpi.com/article/10.3390/v15020472/s1, Table S1. PRISMA 2020 check list; Table S2. Characteristics of 39 studies included in the meta-analysis of prevalence of avian influenza viruses in wild birds in South Korea; Table S3. Characteristics of 10 studies included in the meta-analysis of seroprevalence of avian influenza viruses in wild birds in South Korea.
