# Supplementary material for: Prevalence, Seroprevalence and Risk Factors of Avian Influenza in Wild Bird Populations in Korea: A Systematic Review and Meta-Analysis
Source: Viruses. 2023 Feb 8;15(2):472. doi: 10.3390/v15020472 (PMC9958818; doi:10.3390/v15020472)
Supplement: Supplementary file 1 [file viruses-15-00472-s001.zip › Table S2.pdf]

Table S2: Characteristics of 39 studies included in the meta-analysis of prevalence of avian influenza virus in wild birds in South Korea.

Author/Year	Publication status	Sampling season	Sample size	Positive samples	Sample type	Detection method	Bird species
Baek at al. 2021	Published	NR	13033	75	mixed	RT-PCR	NR
Cheon at al. 2018	Published	NR	52693	413	feces	HA-test	NR
Kim et al. 2015	Published	NR	771	167	carcass	RT-PCR	NR
Kim et al. 2016	Published	Fall/Winter	27	19	carcass	RT-PCR	Anseriformes
Kim et al. 2019	Published	Fall/Winter	11145	124	feces	rRT-PCR	NR
Kim et al. 2021	Published	NR	16317	445	feces	RT-PCR	NR
Kwon et al. 2017	Published	Fall/Winter	50	1	feces	RT-PCR	NR
Kwon et al. 2017	Published	Fall/Winter	391	1	feces	virus isolation	NR
Kwon et al. 2018	Published	Fall/Winter	453	40	feces	RT-PCR	NR
Kang et al. 2010	Published	NR	28214	225	feces	RT-PCR	NR
Jeong et al. 2014	Published	NR	9984	10	cloacal swab	RT-PCR	Anseriformes, Charadriiformes, NR
Jung 2018	Non-published	Fall/Winter	2184	24	feces	RT-PCR	Anseriformes, NR
Lee et al. 2010	Published	Fall/Winter	743	35	feces	virus isolation	Anseriformes
Lee et al. 2010	Published	Fall/Winter	1721	16	feces	rRT-PCR	NR
Lee et al. 2011	Published	Fall/Winter	728	16	feces	rRT-PCR	NR

Lee et al. 2017	Published	NR	31146	202	feces, cloacal swab	RT-PCR	NR
Lee et al. 2018	Published	NR	74162	590	NR	RT-PCR	NR
Lee et al. 2020	Published	NR	15001	180	NR	RT-PCR	NR
Nam et al. 2011	Published	Fall/Winter	350	16	feces	RT-PCR	NR
Na et al. 2021	Published	Fall/Winter	2499	2	feces	rRT-PCR	NR
Nguyen et al. 2021	Published	Fall/Winter	500	30	feces	RT-PCR	NR
Oh et al. 2018	Published	NR	38921	123	feces	RT-PCR	NR
Shin et al. 2015	Published	Fall/Winter	20508	1497	feces, carcass, cloacal swab	RT-PCR	Anseriformes, Pelecaniformes, NR
Yeo at al. 2021	Published	NR	500	30	feces	HA-test	NR
Yeo at al. 2019	Published	Fall/Winter	500	1	feces	RT-PCR	NR
Na 2020	Non-published	Fall/Winter	3256	28	feces	rRT-PCR	NR
Nam 2019	Non-published	NR	14720	201	feces	RT-PCR	NR
Kang 2016	Non-published	NR	13386	103	Feces	RT-PCR	NR
Shin 2016	Non-published	NR	44078	1624	Feces, carcass, cloacal swab	RT-PCR	Anseriformes, Charadriiformes, Gaviiformes,

							Passeriformes, Strigiformes, Gruiformes, Columbiformes, Ciconiiformes, NR
NIER 2012	Non-published	NR	11128	522	feces	RT-PCR	NR
					Carcass, cloacal swab	HA-test	Anseriformes, Gruiformes, Passeriformes
NIER 2013	Non-published	NR	11007	493	feces	RT-PCR	Columbiformes
					cloacal swab, carcass	HA-test	Anseriformes, Columbiformes, Passeriformes, NR
NIER 2014	Non-published	NR	10485	463	feces	RT-PCR	NR
NIER 2016	Non-published	NR	27643	111	Feces, carcass, cloacal swab	RT-PCR	NR
EWU 2007	Non-published	Fall/Winter	740	8	Feces, carcass	RT-PCR	Anseriformes, Galliformes, Columbidae, Passeriformes, Piciformes, Pelecaniformes, Galliformes
NIER 2011	Non-published	NR	17784	181	feces, cloacal swab, carcass	RT-PCR	Anseriformes, Charadriiformes, Passeriformes, Columbidae, Cuculiformes, NR
NIER 2015	Non-published	NR	15566	796	Feces, cloacal swab	RT-PCR	NR

APQA 2006	Non-published	NR	689	3	cloacal swab	HA-test	Anseriformes, Charadriiformes, Accipitriformes, Strigiformes, Pelecaniformes, Piciformes, Accipitriformes, NR
APQA 2019	Non-published	NR	20219	178	cloacal swab	HA-test	Anseriformes, Charadriiformes, Passeriformes, Columbiformes, Gruiformes, Pelecaniformes, Piciformes, Coraciiformes, Strigiformes, Caprimulgiformes, Accipitriformes, Galliformes, NR
APQA 2018	Non-published	NR	92587	736	Feces, carcass	HA-test	NR

Abbreviations: NIER: National Institute of Environmental Research; EWU: Ehwa womans university; APQA: Animal and Plant Quarantine Agency; NR: Not reported; RT-PCR: reverse transcription polymerase chain reaction; HA-test: Hemagglutination test.
