# Supplementary material for: Prevalence, Seroprevalence and Risk Factors of Avian Influenza in Wild Bird Populations in Korea: A Systematic Review and Meta-Analysis
Source: Viruses. 2023 Feb 8;15(2):472. doi: 10.3390/v15020472 (PMC9958818; doi:10.3390/v15020472)
Supplement: Supplementary file 1 [file Table S3.pdf › Table S3.pdf]

Table S3: Characteristics of 10 studies included in the meta-analysis of seroprevalence of avian influenza virus in wild birds in South Korea.

Author/Year	Publication status	Sampling season	Sample size	Positive samples	Detection method	Bird species
Kim et al. 2018	Published	NR	238	17	ELISA	Strigiformes, Pelecaniformes, Anseriformes, Charadriiformes, Passeriformes, Gruiformes, Gaviiformes, Galliformes, Falconiforme
Jeong et al. 2014	Published	NR	9984	202	HI-test	Anseriformes, Charadriiformes, NR
Shin et al. 2015	Published	Fall/Winter	72	29	ELISA	Anseriformes, Gruiformes
Shin 2016	Non-published	NR	2248	777	ELISA	Anseriformes, Charadriiformes, Columbiformes, Ciconiiformes
NIER 2012	Non-published	Fall/Winter	891	440	ELISA	Anseriformes, Gruiformes
NIER 2013	Non-published	Fall/Winter	854	195	ELISA	Anseriformes
NIER 2014	Non-published	NR	679	256	ELISA	Anseriformes, Pelecaniformes
NIER 2011	Non-published	NR	1229	14	HI-test	NR
NIER 2015	Non-published	NR	717	325	ELISA	Anseriformes, Charadriiformes, Pelecaniformes
APQA 2019	Non-published	All season	20118	1182	HI-test	Accipitriformes, Anseriformes, Caprimulgiformes, Charadriiformes, Columbiformes, Coraciiformes, Galliformes, Gruiformes, Passeriformes, Pelecaniformes, Piciformes, Strigiformes, NR

Abbreviations: NIER: National Institute of Environmental Research; APQA: Animal and Plant Quarantine Agency; NR: Not reported; HI-test: Hemagglutination Inhibition test, ELISA: Enzyme-linked immunosorbent assay.
